# BLocate: A Building Identification Scheme in GPS Denied Environments Using Smartphone Sensors

**DOI:** 10.3390/s18113862

**Published:** 2018-11-09

**Authors:** Imran Ashraf, Soojung Hur, Yongwan Park

**Affiliations:** Information and Communication Engineering, Yeungnam University, Gyeongbuk, Gyeongsan 38541, Korea; ashrafimran@live.com (I.A.); sjheo@ynu.ac.kr (S.H.)

**Keywords:** building identification, magnetic field, smartphone sensors, ANN, global positioning system, accelerometer

## Abstract

Indoor localization systems assume that the user’s current building is known by the GPS (Global Positioning System). However, such assumptions do not hold true in GPS denied environments or where the GPS cannot determine the user’s definite location. We present a novel solution to identify the building where the user is present now. The proposed building identification method works on the pervasive magnetic field using a smartphone. The accelerometer data determines the user’s activity of being stationary or walking. An Artificial Neural Network is used to identify the user’s activities and it shows good results. The magnetometer data is used to identify the user’s current building using the fingerprinting approach. Contrary to a traditional fingerprinting approach which stores intensity values, we utilize the patterns formed by the magnetic field strength in the form of a Binary Grid (BG). The BG approach overcomes the limitation of Dynamic Time Warping (DTW) whose performance is degraded when the magnitude of the magnetic data is changed. The experiments are performed with Samsung Galaxy S8 for eight various buildings with different altitudes and number of floors in Yeungnam University, Korea. The results demonstrate that the proposed building identification method can potentially be deployed for building identification. The precision, UAR (Unweighted Average Recall), F score, and Cohen’s Kappa values are used to determine the performance of the proposed system. The proposed systems shows very promising results. The system operates without any aid from any infrastructure dependent technologies like GPS or WiFi. Furthermore, we performed many experiments to investigate the impact of isolated points data to build fingerprint database on system’s accuracy with 1 m and 2 m distance. Results illustrate that by trading off a minor accuracy, survey labor can be reduced by 50 percent.

## 1. Introduction

In the modern era of ubiquitous computing where the demand for Location Based Services (LBS) is increasing exponentially, positioning requires two fundamental characteristics: pervasive coverage and infrastructure independence. Precise location determination plays a very crucial role, not only for LBS but also for health care, assets monitoring, emergency search, rescue response, etc. Outdoor localization is mostly based on the GPS technology as it can provide an estimated location as accurate as 5 m. However, in many situations where the user location is surrounded by high-rise buildings or cloudy environments, the positioning accuracy can drop drastically to as low as 50 to 76 meters [1,2]. GPS, as well as Russian GLONASS constellation, provide similar positioning accuracy [3]. However, for the indoor environment, it does not fulfill the first requirement of being pervasive due to the signal attenuation caused by reflection and refraction, and lower signal-to-noise ratio (SNR).

The researchers spotlighted other emerging technologies that can be used for positioning, especially in the indoor, and GPS denied environments. The primary focus of such research is to utilize the modern smartphones which are pervasive and infrastructure independent. Unlike RFID (Radio Frequency ID), IR (Infrared), iBeacons, Ultrasound, vision, and Wi-Fi based indoor positioning systems, smartphone based systems use built-in sensors of smartphones [4]. RFID, Wi-Fi, and Bluetooth based systems are called infrastructure dependent systems as they require additional hardware to perform localization. Infrastructure dependence in this paper refers to the hardware infrastructure without which the positioning and localization systems cannot work.

The smartphone based systems are cheap as the installation and maintenance of additional infrastructure is not needed. One limitation of these systems is their dependence on GPS to know the place/building information before indoor positioning can be performed. Many such techniques consider that the starting position is known as a priori or consider a naive approach that the GPS calculated position of a building is correct where the user currently is, before localizing it indoors. The building identification is an important element of localization systems which perform both indoor and outdoor localization. GPS accuracy to determine the user’s current building is limited as stated above. Many current indoor localization systems operate on the assumption that the correct building information is already known. Thus, the localization is performed with respect to the known building, e.g., in the case of the Wi-Fi based system, the map of the current building and the database is loaded to perform the localization. The correct building information of the user plays a pivotal role in such localization systems. Thus, many research works have proposed the integration of GPS with other sensors [5,6]. However, the problem is the uncertainty of GPS satellites, which is caused mainly by the signal fading. The signal fading on account of the canyons and closely constructed multi-structural buildings can hinder GPS positioning accuracy. Similarly, semi-outdoor environment affects GPS performance and can wrongly localize a pedestrian to a wrong building. Additionally, GPS localization is unreliable and can be inaccurate when the signal becomes weak e.g., in the proximity of tall buildings [7]. Moreover, GPS sensors become erroneous and uncertain in the case of sporadic unavailability of the satellite signals [8]. Many indoor localization schemes simply assume that a clear pre-knowledge on the building information has been known, but such assumptions hardly remain valid in real scenarios.

We propose the use of the magnetic field data to identify the building where the user is currently walking. The magnetic sensor in the smartphone provides magnetic *x*, *y*, and *z* values that can be used for this purpose. The earth’s uniform magnetic field within a small constrained area is disturbed by the presence of ferromagnetic materials like elevators, door, and concrete structures and causes direction anomalies. However, such disturbances are researched to serve as a unique signature also called the fingerprint [9] and can be leveraged to identify different buildings. The magnetic field has some inherent limitations, e.g., varying magnetic attitude where different devices show different values for the same magnetic field [10] as well as different values by the same device during the different time of the day. Similarly, low discernibility (strength of magnetic field is very weak, i.e., in few μT) and sensitivity (magnetic field reading is affected by indoor infrastructure) also need to be considered. However, in spite of such limitations, many localization systems [11,12,13,14,15,16] have been proposed, which utilize the magnetic field for localization.

This research presents a novel technique which makes the use of the smartphone magnetometer for building identification and, to the best of authors’ knowledge, it is the very first attempt to utilize the magnetic field to identify buildings. The contributions of this research include an infrastructure free building identification model which solely works on smartphone sensors and a deep learning based ANN model which can predict a user’s activity of walking and stationary. Additionally, we use the Binary Grid (BG) to prepare the fingerprint database which stores the patterns formed by the magnetic field instead of magnetic magnitude itself. This overcomes the limitation of Dynamic Time Warping (DTW) whose performance is affected by the varying magnitude of magnetic data collected over different time.

The rest of the paper is organized in five sections. Section 2 describes the related work. Section 3 illustrates the proposed method and its working mechanism. The experiment setup and results are discussed in Section 4 while discussions and the conclusion are given in Section 5 and Section 6, respectively.

## 2. Related Work

The building identification is not a new problem and has been studied for quite a long time. GPS is considered the chief technology for building localization and it works quite fine. Besides GPS, other assisting technologies including cameras and most recently barometers are used to achieve a higher accuracy.

The authors in [5] presented a method to identify a building using a smartphone camera, local 2D Google maps, and GPS. A partial top view of the building, orientation, and distance relative to the camera are fed as inputs to a probability model which is based on kernel density estimation. In addition, it also utilizes smartphone accelerometer and magnetic sensors to estimate the camera ray directions as input features. The results indicate that the proposed system achieves an accuracy of 92.7% to correctly identify the buildings with a processing time of approximately six seconds for 87% of the cases.

The authors in [17] propose an approach for large area classification using a WiFi fingerprint based method. The approach determines whether the user to be localized is indoors in the area for which a radio map is already built or outdoors in the vicinity. The study includes the investigation of the accuracy of several techniques for classification including support vector data description (SVDD), self-organizing map (SOM), minimax probability machine (MPM) and principal component analysis (PCA). The results show that PCA can achieve a classification accuracy of 95.69%. The authors in [18] present a method which utilizes the SNR of GPS to locate a user near the building entrances. The SNR of GPS drops closer to the building walls while GPS provides the accurate location information for a short period of time before entering into the buildings. This transition time is used to find the most probable building location of the user. The technique makes the use of GPS satellites behind the user and at high altitudes to this end and asserts that such satellites can provide more reliable information. The research work also confirms that the accuracy of the GPS localization is affected near the buildings, especially, when LOS (line of sight) with GPS satellites is lost.

The authors in [19] propose the fusion of GPS signals with smartphone camera images to complement the deficiencies of each technique. The approach is based on a bags of words (BOW) approach which utilizes the important features selected from camera images to rectify the positioning error caused by GPS noisy signals. Both scenarios, where GPS signals correct the visual localization and vision-based localization to improve the GPS localization, are studied. The results show that the proposed method can perform the localization within a 7.5 m distance. The authors in [20] present a method to mitigate the error of GPS position estimates in the urban areas. The proposed approach reduces the effect of signal reflection from NLOS (non-line-of-sight) satellites. The approach utilizes the publicly available 3D models but re-calculates the heights using the smartphone camera. Using 3D models and height information, the model estimates the path inflation caused by the signal reflection and re-estimates the calculated location. The adopted method is able to reduce the localization error by 6 to 8 m.

Several similar research works focus on IO (indoor-outdoor) environment detection as it is one of the important tasks in the active field of indoor positioning and localization. The majority of existing research works on indoor localization operates on the assumption that the user is in an indoor environment already when they start to locate him indoors. However, this poses challenges in scenarios where the localization environment contains both the indoor and outdoor environments, like the open covered space between two buildings and over bridge open corridors, etc. Thus, building identification is also very important in such situations where the positioning system needs contextual status to switch between indoor and outdoor positioning modules. The authors in [21] propose a system for indoor–outdoor detection by leveraging the onboard Bluetooth module. Sparse low power Bluetooth devices are installed at the specified landmarks at intermediate regions e.g., covered corridors and entry/exit points. The conducted experiments reveal that the system achieves higher accuracy especially for semi-outdoor environments and consumes less battery than the existing schemes. The system achieves a localization accuracy of 10 m at 90 percent.

The authors in [22] use Bluetooth signals to detect the user indoor and outdoor location. The underlying fact about the GPS accuracy that it is good outdoors and worse indoors, is used in an inverse way for the Bluetooth. Thus, when the distance estimated from Bluetooth is larger, the user is outdoors and vice versa. The experimental results show good indoor–outdoor switching as compared to the GPS. The authors in [23] present an IO detector which is based on four various components including a signal serving cell tower, Wi-Fi, activity recognition, and light intensity captured from the smartphone. It makes the use of these modules and can discriminate four different areas of indoor, outdoor, urban, and complex environments. The experimental results demonstrate that the proposed system outperforms GPS in all four of the test environments. The discussed research works perform well in certain specific environments; however, they are limited in functionality on account of certain factors. First, most of the listed methods rely heavily on GPS, so the inaccessibility of GPS severely limits their functionality. Secondly, other similar systems pertain to additional installation, e.g., Bluetooth or Wi-Fi based systems that make them vulnerable and incur additional cost as well. Additionally, GPS based systems are power-hungry and consume more batteries. This research aims to present a solution which neither utilizes GPS nor necessitates any additional infrastructure. It rather makes use of the built-in sensors which are already made available in the smartphones.

## 3. The Proposed Method

**Figure 1:**
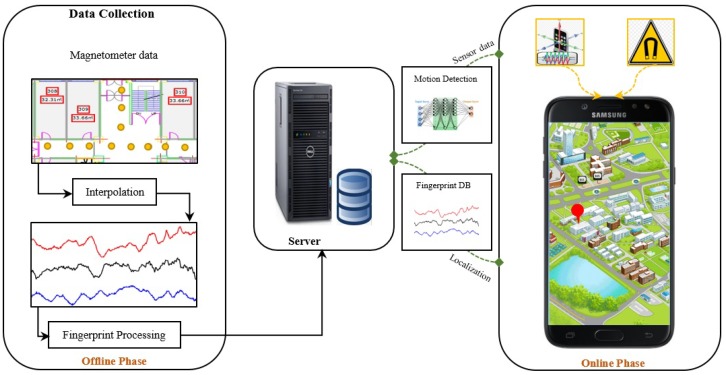
The diagram of the proposed localization scheme.

The proposed building identification method utilizes the built-in sensors of the smartphone. Figure 1 shows the block diagram of the proposed system. Two built-in sensors including accelerometer and magnetometer are utilized in the proposed method. The accelerometer data is used to identify whether the user is walking or stationary. On the other hand, the magnetometer data is utilized to identify the building where the user currently is.

The fingerprint database is built using the magnetometer data for this purpose. The concept of the fingerprint is to connect a fingerprint/signature to relevant locations in a given indoor environment. The fingerprint represents a feature like signal strength, a pattern of a signal or any other similar characteristic which makes it possible to distinguish between various locations. Analogous to the Wi-Fi fingerprinting where RSSI (Received Signal Strength Indicator) [10] serves as a fingerprint, in magnetic fingerprinting, the magnetic flux density, also referred to as the magnetic field strength, is used as a fingerprint. The area is divided into grid points separated by a distance of 1 meter. A total of 100 samples are collected at each point and their normalized value serves as the fingerprint for the associated location. There are two possibilities for a user to be in a specific building:

(i) The user is entering a specific building now.
(ii) The user is already in a building.

**Figure 2:**
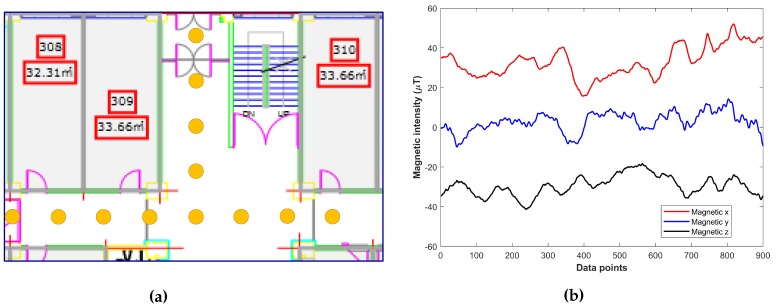
(**a**) data collection at indexed locations; (**b**) interpolated magnetic data after data collection.

For the first option, the building identification can be performed by making a fingerprint database of only the portion close to the entrances. Since we use only 4 sec data, we need to collect the fingerprints of only a 4 to 6 meter area, starting from the entrances. However, the second scenario requires building the database for the complete building, as we do not know where the user is exactly right now. Thus, keeping these two possibilities in mind, we make the fingerprint database of the whole building. Since during the building identification the magnetic data is collected while the user is walking, we need the fingerprints between the selected location points as well. The spline interpolation is applied to generate the intermittent values between the grid points. The fingerprint collection process and the resulting interpolated values are shown in Figure 2.

Traditional magnetic fingerprint-based systems use the magnetic intensity as the fingerprint and thus have two major limitations. First, the magnetic intensity value may be different during different collection times even with the same device, which limits the performance. Second, various devices show different magnetic intensity even for the same location, which leads to device dependence. These issues are handled by using the magnetic field patterns in [24]. The magnetic field intensity is transformed into magnetic patterns for this purpose. The patterns are stored as a matrix called Binary Grid (BG) and Algorithm 1 is used for that purpose. The BG contains 1’s and 0’s only; 1 where a magnetic field value is present and 0 otherwise. The pattern forming procedure is added here for completeness.

**Algorithm 1** Generate the magnetic pattern
1:
$max_{v}=max\left(G_{w}\right);$
2:
$min_{v}=min\left(G_{w}\right);$
3:
M⟵length(f);
4:
$N⟵round(max_{v}-min_{v})+1;$
5:
$\mathrm{Define}\;B_{m}\;\mathrm{of}\;N\;\times \;M\;\mathrm{order};$
6:
**for** j⟵toM **do**
7:    $r⟵round(max_{v}-G_{w}\left(j\right))+1;$8:    $B_{m}\left(r,j\right)⟵1;$9:
**end for**



**Figure 3:**
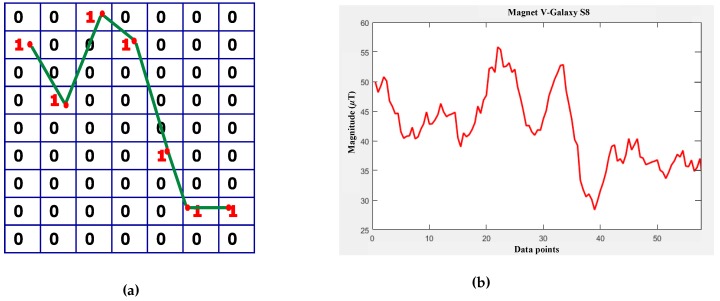
(**a**) Sample binary grid for first 10 data points; (**b**) magnetic field data.

Let’s assume that the magnetic values shown in Figure 3 are to be transformed into a magnetic pattern. First, a BG is declared with columns equal to the length of the magnetic data. The number of rows in the grid is the difference between the maximum and minimum magnitude of the given magnetic data. First, a matrix containing zeros of N×M size is declared where *N* shows the number of rows and *M* is the number of columns. Then, each magnetic data point is taken and converted to a row number by subtracting it from the maximum magnitude of data and rounding it. Afterwards, a 1 is added to the calculated row and this process is repeated for all the data values. The BG shown in Figure 3a is for the first ten values only of the graph shown in Figure 3b.

**Figure 4:**
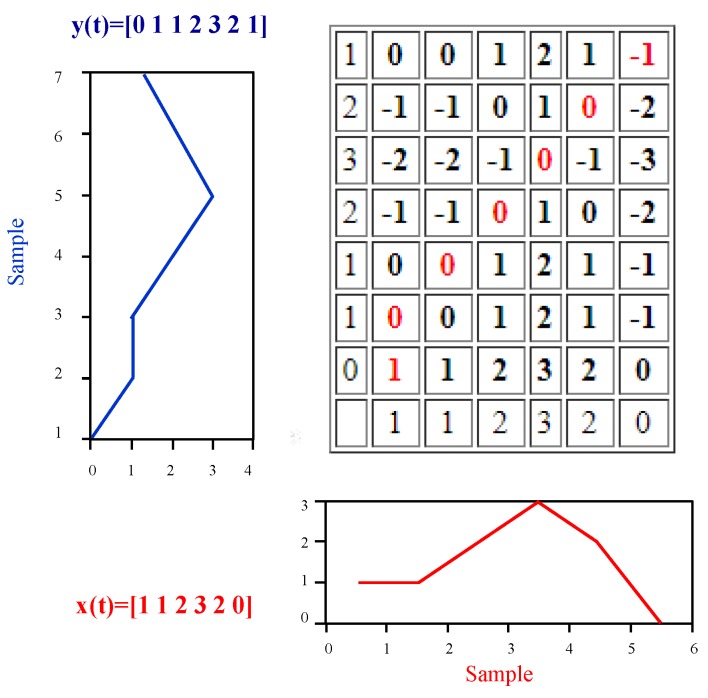
The process of dynamic time warping.

The matching between the user collected samples and magnetic pattern database is performed using the Dynamic Time Warping (DTW). The DTW was first proposed by Berndt and Clifford in 1994 [25]. It was originally designed for speech recognition systems; however, it was later adopted by other domains as well. The DTW is a similarity measure that is used to calculate the distance between two time series. A time series is a sequence of measurement data over time. The DTW is used to measure the similarity for time series where the varying speed of the data collection source may result in a different length of the measured data. Similarly, the shape (magnitude) of the measured data may also change due to the speed, which makes it difficult for simple distance measuring methods to find the similarity. Simple distance measuring techniques are one-to-one linear alignment techniques while the DTW is one-to-many non-linear alignment, which makes it possible to find the similarity between the samples of different length. Consider that the $X=\{x_{1},x_{2},\ldots ,x_{n}\}$ and $Y=\{y_{1},y_{2},\ldots ,y_{n}\}$ are two time series that we want to match. A warping path *W* shows a contiguous set of matrix elements which defines a mapping between the given elements of *X* and *Y*. The warping path is built by arranging the elements of *X* and *Y* on the sides of a grid as shown in Figure 4. Both sequences start from the bottom left of the grid.

**Figure 5:**
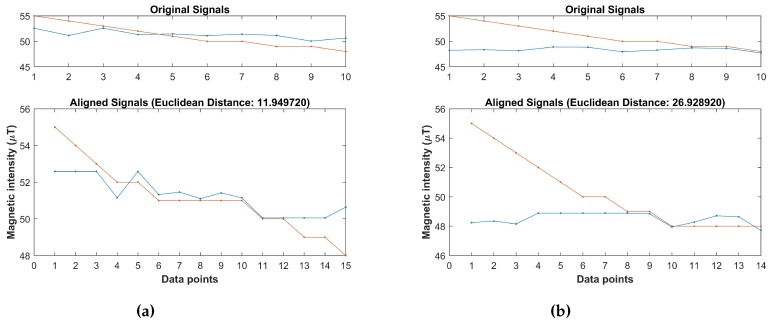
The distance of two samples of the same location during the different time. (**a**) Euclidean distance for sample 1; (**b**) Euclidean distance for sample 2.

Each cell of the grid shows the distance between the elements of two sequences. The warping path is through the cells that show the minimum distance. The overall distance represents the minimum of the sum of distances between the individual elements of given sequences. The DTW technique is good at comparing the sequences of varying lengths, yet it compares the magnitude of the given sequences. This limitation severely confines the systems which are based on the magnetic field data. The reason is the varying magnitude of the magnetic data. For example, Figure 5 shows the magnetic data samples collected during the different time of the day with the same smartphone and the calculated distance using the DTW. It shows the Euclidean distance of 11.94 and 26.92 for the samples of the same place. Thus, although the samples are for the same location taken during different collection time, the distance is very different.

**Figure 6:**
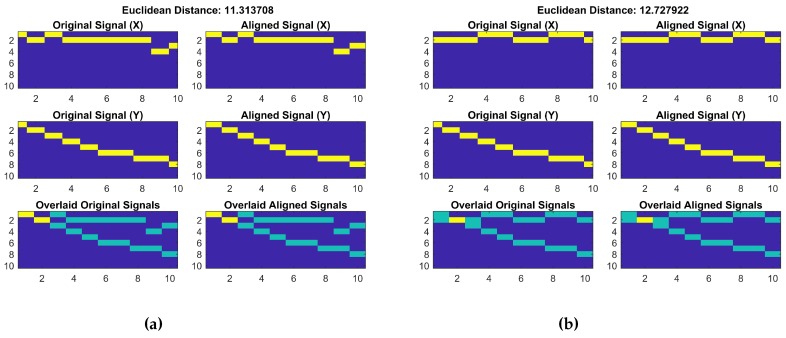
The distance between the magnetic patterns over different time. (**a**) Euclidean distance for pattern 1; (**b**) Euclidean distance for pattern 2.

We already mentioned that the magnetic data for a given location may show different magnitudes every time we collect the data. Thus, if we use traditional DTW, it will produce erroneous results. Experiments reveal that the magnetic data magnitude may change; however, the patterns of magnetic data remain almost similar. Thus, we utilize the patterns formed by the magnetic data. Figure 6 shows the distance calculated by using the DTW for the magnetic patterns and the fingerprint database. It is also important to mention that the fingerprint database also contains the magnetic patterns now. The magnetic pattern fingerprint database is built using Algorithm 1 discussed above.

The Euclidean distance calculated using the magnetic pattern of the given samples are very similar. Contrary to the previously calculated distance of 11.94 and 26.92, now the distance is 11.31 and 12.72 for the given samples. It shows that, when the DTW is used with the BG of magnetic patterns, it becomes more accurate and less prone to the changes in the magnitude of magnetic field data. The data collected from the magnetic sensor is used to make the fingerprint database while the building identification process uses the data from the accelerometer and magnetometer. The data from these sensors are collected over 4 sec and split into four frames each containing 1 sec data. Each frame is processed separately and the final decision is made after the processing of four frames is completed.

### 3.1. The Accelerometer

**Figure 7:**
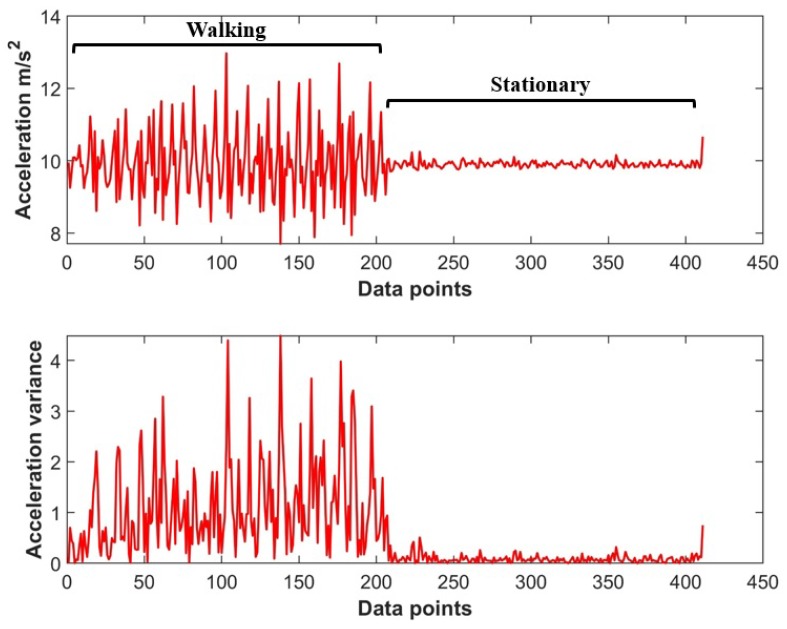
Acceleration data for walking and stationary states.

The accelerometer is used to identify the user’s state of walking or stationary in a given time frame *f*. The size of a frame is 1 sec and it contains 10 measured values of acceleration data along *x*, *y*, and *z*-axis as the data is collected at a sampling rate of 10 Hz (new measurement after 100 ms). Using the given three axes of accelerometer, the total acceleration is calculated as:

(1) $$a_{i}=\sqrt{a_{x_{i}}^{2}+a_{y_{i}}^{2}+a_{z_{i}}^{2}},$$

where *i* shows the measurement for the given frame *f*. We want to use the acceleration magnitude to detect if the user is walking. The acceleration data as shown in Figure 7 is very different for the user’s activities of walking and being stationary.

A simple threshold-based movement detection can also be used to identify if the user is walking or standing stationary. However, the limitation of using the threshold-based heuristic approach is its low accuracy in many cases. Occasionally, phone shaking during the stationary mode generates the acceleration and the data crosses the threshold set to detect the user walking state. The ANN (Artificial Neural Network) identifies the sequence patterns formed while walking and its accuracy is higher than that of a simple threshold-based movement detection. The ANN has shown good performance even in such cases. We use an ANN for the classification of user activities as many types of research [26,27,28] have utilized ANN for similar tasks and showed good classification results. The correct classification of user’s activity of walking is very important for the proposed method. The magnetic patterns need to be collected while the user is walking as when standing stationary there is no change in the magnetic field data. Thus, the magnetic pattern is a straight line as a result which will affect the overall accuracy of building identification.

The ANN is the mathematical modeling of human brain which imitates the function of brain neurons. Similar to a neuron, it is comprised of three layers: an input layer, an output layer and hidden layer(s). Edges are used to connect these layers and each edge bears a weight. Edge weights are determined during the process called back-propagation. An important process in ANN modeling is the selection of the appropriate feature set. Thus, a feature importance analysis is desirable before features are used for training. The Repetitive Feature Elimination (RFE) is performed for the current study to select the most suitable features. The RFE recursively eliminates less important features, and builds the prediction model on those features only, which remain present after the specified iterations. We use a set of eight features including *x*, *y*, *z*, and total acceleration, and variance of *x*, *y*, *z*, and total acceleration. The variance of acceleration in *x*, *y*, *z*, and total acceleration have higher importance so they are used in the ANN model. The Soft-max activation function is used for ANN. A total of 3000 samples of each feature are used for supervised training. The data is collected at a sampling frequency of 10 Hz and a low pass filter is used in the pre-processing phase to reduce the effect of noise. The ANN achieves an accuracy of 92.67% with 10,000 samples tested.

### 3.2. The Magnetometer Module

The pivotal part in identifying a building is performed by the magnetometer module as the identification process is totally performed using the data collected by the magnetometer. The correct building is found by matching the collected data against the fingerprint database which is built during the offline phase of the system. The proposed algorithm BLocate is used for localization. Table 1 shows the notations used in algorithm 2 which identifies the buildings.

**Algorithm 2BLocate** Building identification with sensors’ data.
  1:**if** User is walking **then**  2:    **for** j⟵1to4 **do**  3:        $G_{s}\left(j\right)⟵\mathrm{generate}\;\mathrm{Pattern}\;\mathrm{using}\;G_{f};$  4:        **for** k⟵1toN **do**  5:            $E_{bi}^{j}⟵\mathrm{calculate}\;\mathrm{Euclidean}\;\mathrm{Distance}\;\mathrm{using}\;G_{s}\;\mathrm{and}\;dB^{k};$  6:            $mE_{bi}^{j}⟵\mathrm{get}\;\mathrm{minimum}\;\mathrm{Euclidean}\;\mathrm{distance}\;\mathrm{from}\;E_{bi}^{j};$  7:        **end for**   8:        $\tilde{E_{b}^{kN}}⟵\;\mathrm{normalize}\;\mathrm{Euclidean}\;\mathrm{distance}\;\mathrm{using}\;mE_{bi}^{j}\;\mathrm{and}\;N_{b};$  9:        $B_{c}⟵\;\mathrm{get}\;\mathrm{minimum}\;\mathrm{from}\;\tilde{E_{b}^{kN}};$10:    **end for**11:
**end if**
12:$B⟵\;\mathrm{finally}\;\mathrm{localize}\;\mathrm{building}\;\mathrm{by}\;\mathrm{removing}\;\mathrm{outliers}\;\mathrm{using}\;B_{c}$;


The algorithm follows these steps to predict the building where the user is currently walking.

**Step 1**: The data is collected using magnetometer and accelerometer with a smartphone. First of all, the accelerometer data is utilized to classify the user’s mode of walking or stationary. If the user is in a walking state, then the magnetic data is used for further processing.

**Step 2**: The magnetic data is transformed into magnetic patterns. As it is previously mentioned that the magnetic fingerprint database is made up of magnetic patterns during the offline phase, we need to transform the magnetic data to magnetic patterns during the online phase to match them against the fingerprint database.

**Step 3**: During the third step, the online magnetic field pattern is matched against the database of all the floors of all buildings. Since the user’s walking speed varies, it results in different magnetic patterns depending upon the walking speed. We use the DTW for matching as it serves as a tool to compare two time-dependent sequences of varying length [25]. The DTW can become computationally expensive when long sequences are matched. Thus, we take a database sample equal to user sample (1 sec data) and match them to calculate the Euclidean distance:

(2) $$E_{bi}^{j}=\sqrt{{\left(x_{s}-x_{bi}\right)}^{2}+{\left(y_{s}-y_{bi}\right)}^{2}}.$$

**Step 4**: Once the Euclidean distance between the user sample and the fingerprint database is calculated, we need to find the point where the error/distance is the minimum as that point is the most suitable candidate. Thus, each floor’s candidate is the lowest error estimate.

**Step 5**: Once the minimum error for each floor of each building is calculated, these errors are to be normalized. Since each building has a different number of floors and each floor may have a different error depending upon which floor the user is actually on. The magnetic field shows higher similarity to floors of the building where they are taken from. It infers that the magnetic data collected from a floor of a building A should show similar matching results to the other floors of building A as well. Thus, comparing the error value of a single floor is not appropriate. Instead, if we normalize the error with the total number of floors that would give more accurate results. The normalization is done using:

(3) $$\tilde{E_{b}^{kN}}=\sum_{i=1}^{N_{bi}}\frac{mE_{bi}^{k}}{N_{b}},$$

where $mE_{bi}^{k}$ is the minimum Euclidean distance for a floor of the given building and $N_{b}$ represents the number of floors in the given building.

**Step 6**: After normalization, the minimum error value represents the building where the user is right now. As mentioned earlier, the total user collected data is split into four consecutive frames, so this process of building identification is repeated for the four frames. Each frame possesses a vote and the final decision is based upon the votes of the four frames. After this process is complete, we have four predicted buildings which are called building candidates. In certain situations, it is also possible that the predicted buildings are very different so we need to correct the predictions. Since we use four frames data so the output candidates are four buildings and can have one of the following forms:

1. The prediction set has only one building as output.
2. The prediction set has three predictions for one building and one prediction for the other.
3. The prediction set has two predictions each for two different buildings.
4. The prediction set has one prediction for four different buildings.

The first set is very perfect. As the prediction is only one building, there are no outliers. In the second case, the building that has three predictions in the prediction set is selected as the building where the user is positioned. For the third option, where we get two various buildings, the building with lower error is selected as the target buildings. However, if the error is the same as well, then the building with the higher number of floors is the target building. The same procedure is adopted for the fourth case and first priority is given to the candidate with the lower error, and then to the building with a higher number of floors, when each building candidate possesses the same value of error:

(4) $$B=\min_{x\in E_{b}^{kN}}f\left(x\right),\max_{x\in N_{b}}f\left(x\right).$$

### 3.3. Accuracy Measures

We need to understand four basic notations used in the classification before we move forward to calculate the accuracy measures of a classifier [29].

**True Positives** (TP): These represent the positive tuples of a class that are correctly labeled by the classifier.

**True Negatives** (TN): These represent the negative tuples that are correctly labeled by the classifier.

**False Positives** (FP): These are the negative tuples that are incorrectly labeled as positive by the classifier.

**False Negatives** (FN): These are the positive tuples that are incorrectly labeled as negative by the classifier.

Accuracy is a measure widely used to evaluate the performance of a classifier and it is defined in terms of the percentage of the buildings predicted correctly [29]. Using the above described terms of *TP*, *TN*, *FP*, and *FN*, accuracy is calculated using:

(5) $$accuracy=\frac{TP+TN}{TP+TN+FP+FN}\times 100.$$

Precision and recall are two measures which are commonly used to evaluate the performance of a classifier. Precision is a measure which tells what percentage of all tuples are labeled positive that are actually positive. Precision is calculated as:

(6) $$precision=\frac{TP}{TP+FP}.$$

The recall is often referred to as the measure of completeness and it represents the percentage of true positive tuples which are labeled as such. It is also called the sensitivity and calculated as:

(7) $$recall=\frac{TP}{TP+FN}.$$

The *F* score is a statistical analysis measure of classification, which considers both precision and recall of the classifier and computes a score between 0 and 1. The values closer to 1 represent the higher performance of the classifier and *F* reaches 1 when the classifier performs 100% correct classification. The *F* score reflects the average effect of both precision and recall. It is calculated as:

(8) $$F=2\frac{precisioin.recall}{precision+recall}.$$

The Cohen’s kappa coefficient [30] also represented as *k* is a statistic that is used to measure the true inter-rater agreement [31]. It defines the agreement between two evaluators, whereas we define it as the automatic prediction of the building and already defined label of the buildings. It takes the following mathematical form:

(9) $$k=\frac{P_{o}-P_{c}}{1-P_{c}},$$

where $P_{o}$ represents the observed agreements while $P_{c}$ is the portion of the agreement that is expected by chance. There are many interpretations of Kappa value, although no values are universally accepted upon. The authors in [29] state that if the Kappa value is higher than 0.80 it represents the higher strength of agreement and shows that the classification model has high classification accuracy. The values between 0.61 and 0.80 represent good agreement, between 0.41 to 0.60 moderate, between 0.21 to 0.40 fair, while the value lower than 0.20 shows very poor agreement.

## 4. Performance Evaluation

### Experiment Setup

The experiment is conducted in various buildings located in Yeungnam University, Republic of Korea. The buildings are selected with two points in consideration. First, the buildings should have a various number of floors and different indoor infrastructure. Secondly, two groups of buildings are selected including closely located buildings and buildings separated geographically apart by at least 1/2 mile. The goal of this setting is to make the scenario more realistic so that the algorithm’s performance can be evaluated for real applications. Table 2 shows the relevant information of the buildings selected for the experiment.

**Figure 8:**
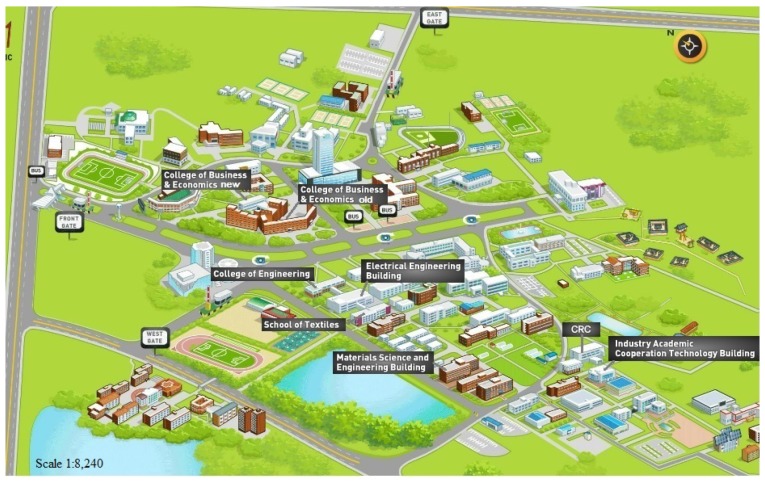
Buildings for the experiment in Yuengnam University.

Figure 8 shows the location of the selected buildings on Yeungnam University’s map. Three buildings including ITE, EE, and TE are connected within a block and they are very close to each other. Similarly BEN and BEO are two different parts of the same building; however, their indoor infrastructure is very different. The BEN is a new building built with iron filled concrete and shows magnetic fluctuations; the BEO, on the other hand, is built with old bricks lacking any concrete or iron thus showing somewhat smooth magnetic attitude. The MSE, CRC, and IACT are separated from each other and have a different number of floors. Few buildings share very similar indoor infrastructure in terms of offices, laboratories and corridor including ME, TE and EE. Thus, the experiment is quite complicated and poses a real challenge.

**Figure 9:**
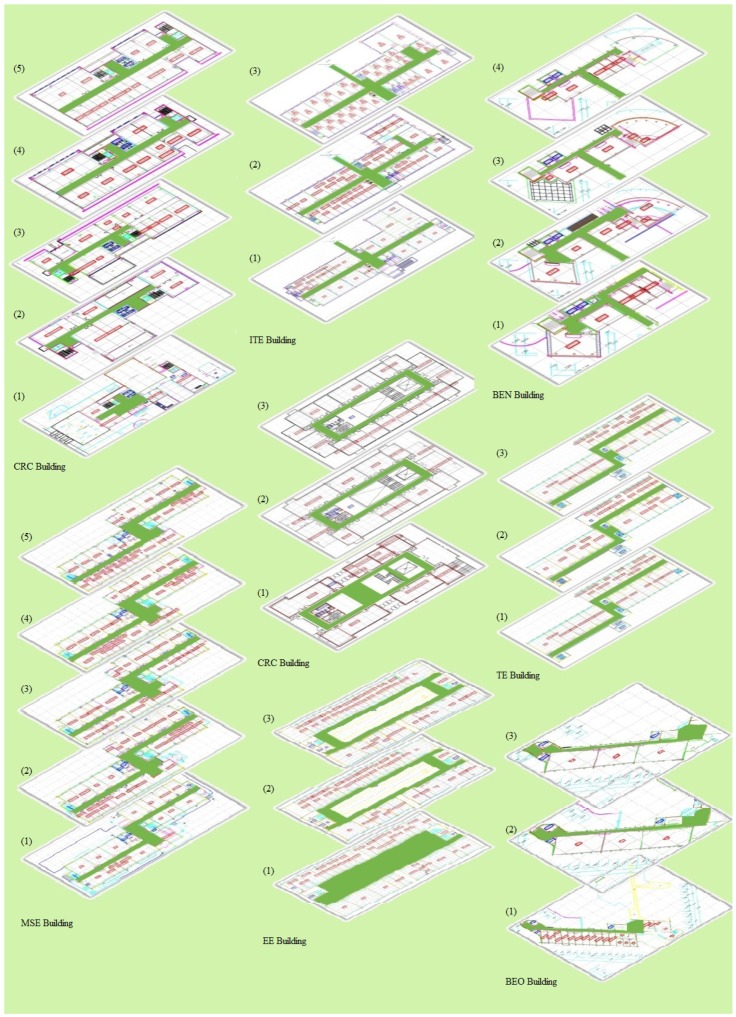
Indoor space of buildings used for the experiment.

The indoor space available for the data collection is different within each building as well as for each floor in some cases. The details of indoor maps of each building and the area used for the experiment is shown in Figure 9. The data is collected at different times of the day on different dates to make the experiment more realistic.

**Figure 10:**
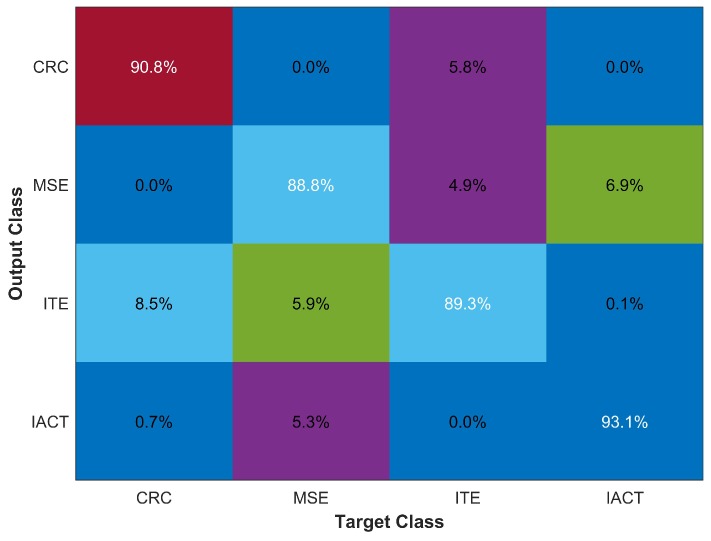
Classification results for four buildings.

Initially, we tested the algorithm with four buildings including IACT, ITE, MSE, and CRC to evaluate its performance with respect to both the response time and accuracy. Figure 10 shows its results for accuracy as well as true positive, true negative, false positive, false negative, F- score and Cohen’s kappa value.

The results show that the algorithm performs very well both for the classification accuracy and the response time. The response time is averaged over 5000 user requests and it includes only the processing time. The processing is done on the server side which is an Intel Core i5 3.2 GHz (Intel, California, USA.) operating on Windows 8.1 (Microsoft Corporation, Washington, USA.) with 8 GB RAM. Table 3 shows the results for other accuracy measures including F score and Kappa etc. A kappa value of 0.86 shows high inter-rater agreement while F score, precision, and recall values also depict the good performance of the proposed method. The average processing time for one user request is 0.21 sec only which involves the processing of 4 sec data from accelerometer and magnetometer. Moreover, the accuracy of 90.54% is excellent as well.

The second experiment is conducted with eight various buildings. The purpose of this experiment is twofold: to analyze how the prediction accuracy is affected and what impact it has on the processing time.

**Figure 11:**
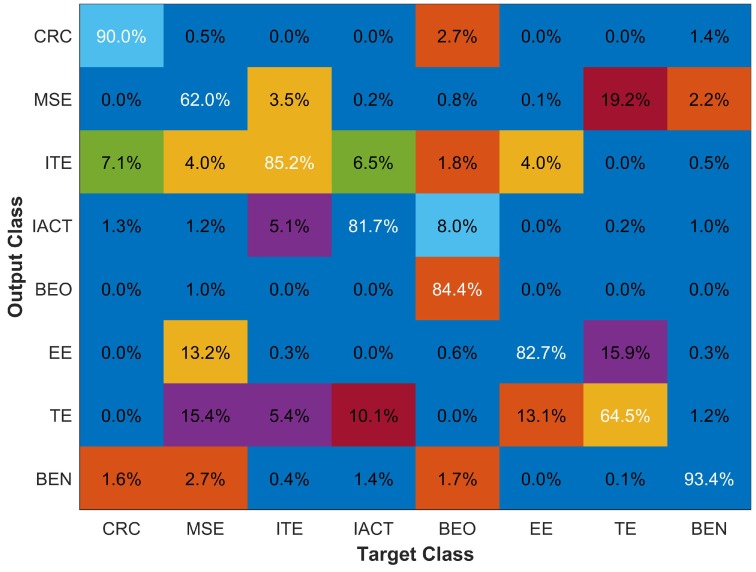
Classification results with eight buildings.

The classification results with eight buildings are shown with a confusion matrix in Figure 11. The classification with eight classes has reduced both the overall accuracy as well as the accuracy of individual classes. The classification accuracy of CRC, ITE, and IACT buildings is reduced by 3.2%, 0.9% and 2.0% only; however, the accuracy of the MSE building is affected severely. Additionally, the average response time has been increased too. The results for other accuracy parameters are given in Table 4.

The reason for an increase in the response time is quite obvious; the higher number of databases led to the increased processing time for matching the user given sample. The rationale for lower performance is that when the number of fingerprint database increases, the probability of databases with similar fingerprint increases likely. This is caused by two factors. First, the closely located buildings may have a similar magnetic field. Second, although still located separately, a similar internal infrastructure may lead to a similar magnetic field. The second reason is more logical and valid because, in university campuses, traditionally many departments are built in an identical internal design which may lead to very similar magnetic behavior. We perform an experiment to check the similarity of magnetic databases with each other. We match each building database to the database of every other building to analyze which buildings exhibit similar magnetic behavior and the results are shown in Table 5.

Results indicate that the magnetic databases of MSE and TE and MSE and EE (shown bold in Table 5) have higher similarity. This explains the lower classification accuracy of MSE (67.7%) and TE (69.9%) as given in Figure 11. It is also shown that the highest misclassification (18.1%) that MSE has is against TE building. We further investigate this by matching floor level databases of these buildings to find which floors of these buildings show similar behavior.

**Figure 12:**
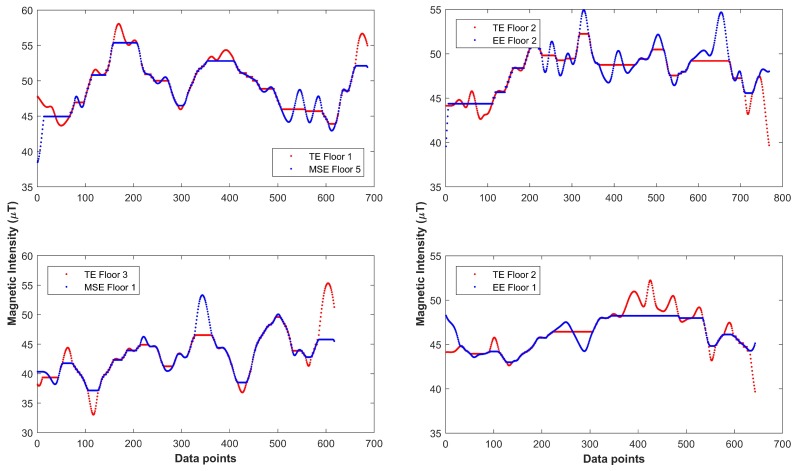
Aligned databases of MSE and TE buildings (**a**); and Aligned databases of TE and EE buildings (**b**).

**Figure 13:**
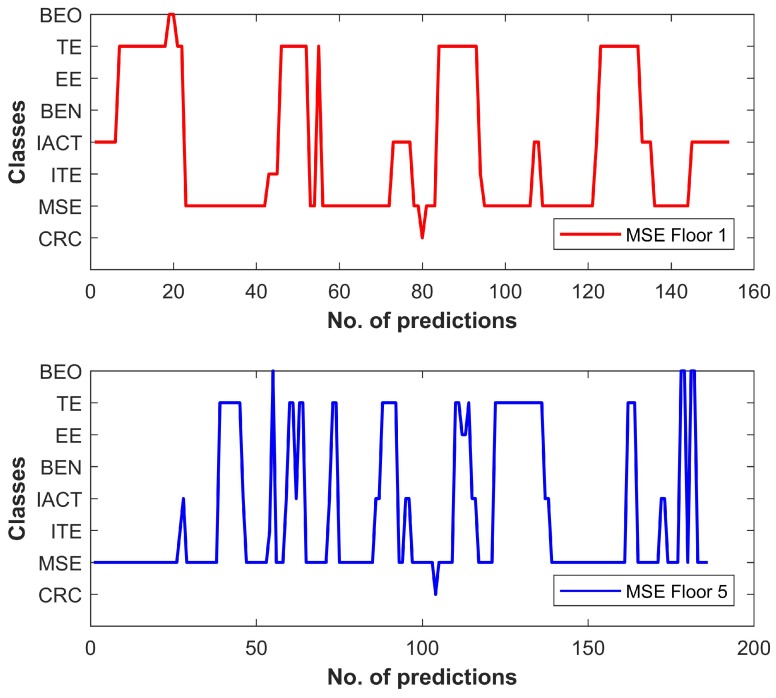
Predictions result using MSE building user data.

Figure 12 shows the results of the comparison between various floor fingerprint databases done using the DTW. The databases are aligned to analyze how much similarities do they have. The results demonstrate that the fingerprint databases of TE floor 1 and MSE floor 5 and TE floor 3 and MSE floor 1 have very identical magnetic patterns. Thus, when we perform pattern matching between the user samples taken from MSE floor 5, it shows more similarity with the TE database than that of MSE at specified locations. As a result, the classification accuracy is decreased. Similarly, the data collected from MSE floor 1 behaves in an identical manner for classification. To further corroborate the above findings, we took additional data from each floor of the MSE building and run a separate classification for validation, and the results are shown in Figure 13.

It further indicates that, when we match the data collected from MSE floor 1 and floor 5, the matching results make wrong predictions associated with TE building as shown in Figure 13. The ground truths for these graphs are MSE floor 1 and floor 5. Only the results for floor 1 and floor 5 are shown because these floors have high misclassification; the prediction for other floors’ data is quite good.

In spite of these incorrect predictions, the presented method performs well for the building identification and achieves an accuracy of 86.37% for all the buildings, while the individual class accuracy can go up to 97.4%. In addition, the average response time is also very nominal, which is only 0.32 sec for one user request. The one critique of the method is that the database fingerprinting process is laborious and time-consuming. However, the databases are built just once and no updating is required unless huge internal infrastructural changes are made. The primary experiment is performed with the database built from the data collected at points separated by 1 m. However, we made experiments with fingerprint databases of buildings which involve data collection at points which are at a distance of 2 m. The purpose is to analyze the changes in the prediction accuracy of the algorithm. The procedure described above is replicated for the experiment with 2 m fingerprint database.

**Figure 14:**
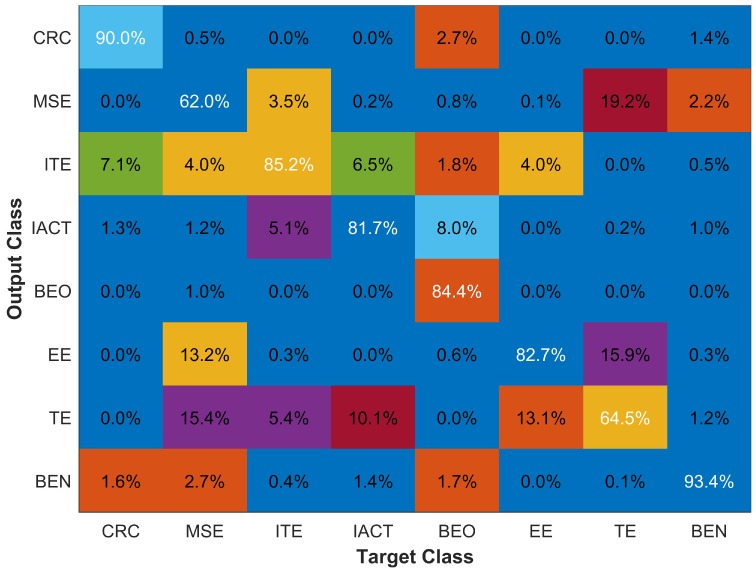
Classification results using a 2 m database with eight buildings.

The results given in Figure 14 demonstrate that, when we use a fingerprint database of 2 m distance, every class suffers a reduction in the classification accuracy, which ultimately causes reduced overall accuracy of the algorithm. The overall accuracy is reduced to 81.47% from 86.37%. The incorrect predictions are primarily resulting from the erroneous pattern matching results. Interpolation generates intermittent values using surrounding points. When data is collected at 2 m distance, the values used for the interpolation are lower, which results in smoothed generated values. The uniqueness of interpolated values is reduced as a result. The results for UAR, and Kappa value for each building are given in Table 6.

**Figure 15:**
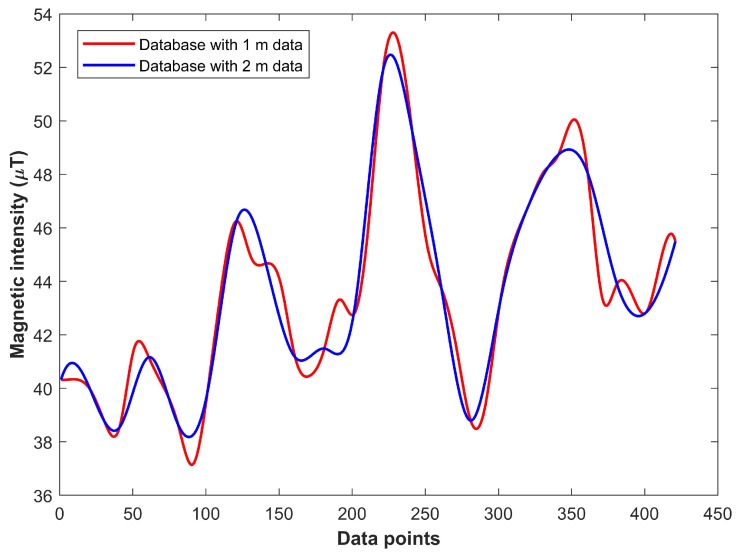
Comparison of databases prepared with 1 m and 2 m separated points.

An auxiliary analysis to validate the above-mentioned notion results in Figure 15. The resulting interpolated values from 2 m data become more even and smooth. This leads to incorrect matching results and lowers the prediction accuracy. Thus, we have to make a trade-off between the surveying labor for the fingerprinting database and accuracy when we consider the fingerprint database.

## 5. Discussion

This study presents an approach to identify the building where a pedestrian is present now. The proposed approach is totally infrastructure free and works without any additional assisting technology. The technique is founded on the built-in sensors of the smartphone and leverages the accelerometer and magnetometer data only. The user collected data is matched against the fingerprint databases of each building of the university departments. The fingerprint databases are pre-built during the surveying phase. However, contrary to a traditional fingerprinting approach which stores magnetic magnitude as the fingerprint, we adopt the Binary Grid (BG) approach which transforms the magnetic magnitude into magnetic patterns and then stores them. The BG approach overcomes the limitation of Dynamic Time Warping (DTW) as well. The DTW approach shows different results for the data of the same place collected during different times. The BG approach followed in this paper shows very similar results for the data collected over different times. The data is collected at points which are 1 m apart and then interpolation is applied to generate the intermittent values between these points. The two important factors associated with a successful model i.e., response time and accuracy are investigated using two scenarios.

The first scenario involves the localization with four buildings which are geographically well separated. The proposed method performs very well both in terms of accuracy and response time and obtains an accuracy of 90.54% and a response time of 0.21 sec, which involves processing 4 sec data gathered from the accelerometer and magnetometer. The second scenario is more realistic where the experiment involves eight buildings with 29 floors in total. In spite of the fact that with an increased number of databases the accuracy can be severely affected, the overall accuracy of the proposed method is 86.37% and the average response time is 0.32 sec only. The accuracy of only two buildings is depraved and lowers under 70%, while other six buildings sustain the accuracy over 80%. In addition, Cohen’s Kappa value for classification with eight buildings is 0.84, which shows the strong inter-rater agreement. Supplementary experiments are carried out to investigate the factors which reduce the accuracy. The fundamental cause of the reduced accuracy is the higher similarity between the fingerprint databases which reduces the classification accuracy in essence. Despite the fact that the results illustrate that the proposed method has huge potential and can be utilized to work in GPS denied environments.

One criticism of the traditional process of fingerprinting approach is the laborious survey that requires both cost and time. Traditionally, during surveys, the data are collected at specified points separated by 1 m; we followed the same procedure. In addition, we, however, performed experiments with a database built using the data collected at points separated by 2 m. The results demonstrate that the overall accuracy is reduced to 81.47% from 86.37%. The elementary reason is the incorrectness of the interpolated values that are generated using the interpolation. The higher the distance between the points used for the interpolation, the more smoothed the interpolated values are that affect the uniqueness of the fingerprints. Thus, the labor for surveys can be reduced, but there is a trade-off between the surveying cost and accuracy.

## 6. Conclusions

Recent indoor localization systems that leverage the smartphone built-in sensors operate on the assumption that the system has former information of the building where the user is currently walking. It is assumed that the system knows the starting position as a priori or use a naive approach that the GPS calculated position is correct. Such assumptions may plot a pedestrian in the building other than where he currently is. Additionally, GPS deprived environments make it very challenging to know the building information for such systems to operate properly. This research presents a building identification scheme which utilizes the pervasive magnetic field to identify the specific building where the user is now. The magnetic patterns based fingerprinting database approach is used and two databases are built with data collected at 1 m and 2 m separated points to investigate the impact of isolated points on the prediction accuracy. The proposed scheme is able to predict accurately at 86.37% considering eight buildings that contain twenty nine floors in total. Similarly, the proposed system is able to accomplish a precision of 0.85, UAR (Unweighted Average Recall) of 0.84, F score of 0.84 and Cohen’s Kappa value of 0.84, respectively, which show high prediction as well as higher inter-rater agreement. The *F* score and Kappa values show very strong agreement between the predicted and labeled buildings. The prediction accuracy of the proposed scheme is 81.47% when we use the fingerprint database with data points which are 2 m apart. However, the system accuracy is reduced by a margin of 4.9%, yet the surveying labor can be reduced by 50% with a 2 m database. The proposed system is totally infrastructure free and leverages the data collected using the smartphone built-in sensors alone. The system utilizes only four frames of magnetometer and accelerometer data collected at 10 Hz. We intend to perform further experimentation with an increased number of buildings and database building using crowdsourcing for public places including the shopping malls in the future.

## Figures and Tables

**Table 1:** Notations used in Algorithm 1.

Notation	Description
A	Set of acceleration data
G	Set of geomagnetic data
f	The length of one frame which is 10 values (1 sec data at 100 ms sampling rate)
B	The building, where currently the user is in
$B_{c}$	The building candidates, where currently the user is in
N	The total number of floors in all buildings
$N_{bi}$	Floor *i* in building *b*
$E_{bi}^{k}$	Set of Euclidean distances between sample *k* and floor *i* of building *b*
m$E_{bi}^{k}$	The minimum of Euclidean distances between sample *k* and floor *i* of building *b*
$\tilde{E_{b}^{k}}$	The normalized Euclidean distance for sample *k* and building *b*

**Table 2:** Buildings’ description used in the experiment.

Complete Name	Abbreviation	Floors
LED-IT	CRC	6
Material Science & Engineering	MSE	5
Information Technology (College of Eng.)	ITE	3
Industry Academic Cooperation Technology	IACT	3
College of Business & Economics-New	BEN	4
Electrical Engineering	EE	3
School of Textile Engineering	TE	3
College of Business & Economics-New	BEO	3

**Table 3:** Accuracy parameters result for four building classifications.

Class	Precision	Recall	F Score
CRC	0.9080	0.8692	0.8882
MSE	0.8883	0.8495	0.8685
ITE	0.8926	0.9181	0.9052
IACT	0.9305	0.9552	0.9427
Overall accuracy		90.54%	
UAR		0.9011	
Kappa Value		0.8586	
Avg. response time		0.21 s	

**Table 4:** Accuracy parameters result for eight building classification.

Class	Precision	Recall	F Score
CRC	0.8764	0.9810	0.9258
MSE	0.6767	0.7958	0.7315
ITE	0.8840	0.9419	0.9121
IACT	0.9115	0.7587	0.8281
BEN	0.9203	1.0000	0.9585
EE	0.8743	0.8370	0.8552
TE	0.6986	0.5173	0.5944
BEO	0.9743	0.9581	0.9661
Overall accuracy		86.37%	
UAR		0.8464	
Kappa Value		0.8421	
Avg. response time		0.32 s	

**Table 5:** The distance between fingerprint databases of all buildings.

	CRC	MSE	ITE	IACT	BEN	EE	TE	BEO
**CRC**	0	5750.96	13396.80	5736.93	4668.80	6611.00	5825.07	6481.80
**MSE**	5750.96	0	2893.87	2586.33	1995.33	**1123.47**	**904.67**	1232.40
**ITE**	13396.80	2893.87	0	3587.44	2569.00	3470.22	3168.44	2772.00
**IACT**	5736.93	2586.33	3587.44	0	1938.08	3677.00	2799.22	2447.33
**BEN**	4668.80	1995.65	25699.00	1938.08	0	2384.00	2095.42	2009.25
**EE**	6611.00	1123.47	3470.22	3677.00	2384.00	0	1393.22	2092.67
**TE**	5825.07	904.67	3168.44	2799.22	2095.42	1393.22	0	1254.33
**BEO**	6481.80	1232.40	2772.00	2447.33	2009.25	2092.67	1254.33	0

**Table 6:** Accuracy parameters result for eight building classification with a 2 m database.

Class	Precision	Recall	F Score
CRC	0.8995	0.9591	0.9284
MSE	0.6198	0.7012	0.6580
ITE	0.8525	0.8955	0.8735
IACT	0.8174	0.7199	0.7656
BEN	0.8441	0.9897	0.9111
EE	0.8274	0.7078	0.7629
TE	0.6452	0.5167	0.5739
BEO	0.9342	0.8840	0.9084
Overall accuracy		81.47%	
UAR		0.7977	
Kappa Value		0.7827	
